# Embedding supportive parenting resources into maternity and early years care pathways: a mixed methods evaluation

**DOI:** 10.1186/s12884-019-2388-2

**Published:** 2019-07-22

**Authors:** Nicola Crossland, Gill Thomson, Victoria Hall Moran

**Affiliations:** Maternal and Infant Nutrition and Nurture Unit, University of Central Lancashire, Preston, PR1 2HE UK

**Keywords:** Breastfeeding, Parenting, Early years, Maternity, Digital health, Mobile phone applications, Health promotion, Health information

## Abstract

**Background:**

During pregnancy and postnatally, women seek information from a variety of sources. The potential to incorporate educational pregnancy and parenting resources into conventional health services is underexplored. In 2014–2016, UK-based charity Best Beginnings used an embedding model to embed three of their resources – the Baby Buddy app, Baby Express magazine, and ‘From Bump to Breastfeeding’ DVD – into maternity and early years care pathways at three sites in the north of England. A mixed-methods evaluation comprising an impact evaluation and a process evaluation was undertaken. Here we report findings from the process evaluation that aimed to understand the embedding process, explore maternity and early years’ professionals’ views and use of the resources, explore women’s engagement with and views of the resources, and identify barriers and facilitators to the embedding process.

**Methods:**

We carried out semi-structured interviews with stakeholders (professionals involved in embedding) and observations of embedding activities to understand how embedding worked. Surveys of postnatal women were conducted over a two-month period both prior to, and after, the resources had been embedded, to ascertain engagement with and views of the resources. A survey of professionals was carried out post-embedding to understand how, where and when the resources were used in practice, and professionals’ views. Descriptive and thematic analyses were undertaken.

**Results:**

Thirty stakeholders took part in interviews. Surveys were completed by 146 professionals, and by 161 and 192 women in the pre and post-embedding phases respectively. Themes derived from analysis of qualitative data were ‘Implementation of the embedding model’, ‘Promotion and distribution of, and engagement with, the resources’, ‘Fit with care pathways’, and ‘Perceptions of the resources’. While survey responses indicated that embedding of the resources into practice was not yet complete, those who had used the resources believed that they had helped increase knowledge, build confidence and support relationship-building.

**Conclusions:**

Incorporating supportive parenting resources into maternity and early years’ care pathways requires a planned embedding approach, committed champions, and senior management support. Findings indicate largely positive views of women and professionals, and suggest the resources can be a beneficial aid for families.

**Electronic supplementary material:**

The online version of this article (10.1186/s12884-019-2388-2) contains supplementary material, which is available to authorized users.

## Background

For most women, the experience of pregnancy, birth, and becoming a mother is a time of significant emotional, physical and social adaptation and adjustment, which can be demanding and at times isolating [1, 2]. Abundant information about pregnancy, birth, infant development and advice about infant care is offered to women by health providers, family, friends, wider society, and commercial sources, but information alone is not necessarily supportive for women, who may have difficulties accessing, understanding or utilising information, or who may feel overwhelmed by the sheer volume of information available [3]. mHealth, the use of mobile communications to deliver health information and services, has gathered much interest in recent years, and there has been vast increase in the number of digital and mHealth applications aimed at pregnant women and new parents [4]. A recent Australian study highlighted the value mothers placed on the information and support they received through digital media, including mobile phone apps [5]. However, a qualitative study by Wilcox and colleagues investigating women’s and professionals’ views of mHealth information sources to support a healthy lifestyle in pregnancy found that although the health professionals envisaged mHealth operating in parallel to conventional services, women were more likely to want mHealth to be integrated into conventional care [7]. Similarly, another recent qualitative study found that pregnant women favoured the integration of eHealth and mHealth applications into usual healthcare [8]. A further study exploring pregnant women’s experiences of using a smartphone app to help manage gestational diabetes reported that women found the app to be a useful source and reminder of information, but that health professionals often did not engage with the app [9].

Most studies to date have investigated the effectiveness of informational tools for pregnant or postnatal women by viewing the intervention as an addition to usual care, and comparing outcomes with those for usual care [6, 10–12]. To date, there are few studies that explore how interventions interact with existing services and what supports their use. One study undertaken in 1998 evaluated the use of evidence-based leaflets intended to support women’s choice in maternity care. In this study, participant-observation research accompanied a cluster randomised controlled trial to understand how staff attitudes and organisational culture influenced the uptake and use of the information leaflets [13]. The evaluation found that while health professionals were largely positive about the principles of giving information, in practice, time pressures and pragmatic concerns limited the extent to which they were used with women [13]. In the UK, the recent National Maternity Review [14] called for NHS providers to invest in accessible e-technological tools to support maternity care; however, the challenge remains for health services as to how to incorporate multimedia tools into care pathways to best support the health and wellbeing of mothers, babies, and families.

Best Beginnings is a UK-based charity whose aim is to improve outcomes and reduce inequalities in child and maternal health, with a special focus on the time period between pre-conception to a child’s third birthday. The charity has created a number of parenting resources to help promote positive health and wellbeing, including a mobile phone app called Baby Buddy, a magazine called Baby Express, and a DVD called From Bump to Breastfeeding. [12] In 2014, Best Beginnings received funding from the UK Department of Health funding to embed these resources within three UK regions, and commission an in-depth evaluation of the embedding process. The evaluation comprised a process evaluation which we present here, and an impact evaluation (presented elsewhere). The process evaluation aimed to explore how and where embedding was undertaken and who was involved, to explore maternity and early years’ professionals’ views of how the resources were being embedded, the extent to which they were used within practice, and to identify barriers and facilitators to the embedding processes.

## Methods

### Study design

We carried out a mixed-methods process evaluation involving: (i) observations of key embedding events; (ii) interviews with stakeholders; (iii) post-embedding survey of maternity and early years’ professionals; (iv) pre- and post-embedding surveys of postnatal women; and (v) a review of data on the numbers of Baby Buddy app registrations supplied by Best Beginnings. The evaluation was carried out between June 2015 and December 2016.

### Study context

The study took place in the north of England, with a participating site from each of three north of England regions (North West, North East, and Yorkshire and Humber). The study was advertised to potential sites in 2015 through the England and Northern Ireland National Infant Feeding Network, the UNICEF UK Baby Friendly Initiative conference, and other conferences relating to maternal and child health. Interested commissioners of maternity/early years services from NHS or Local Authorities (public health) applied to Best Beginnings to be part of the study. Participating sites were required to pay for the supply of Baby Express magazines and From Bump to Breastfeeding DVDs. Commissioners provided information on demographic and organisational variables such as annual birth cohort size, breastfeeding rates, and UNICEF Baby Friendly status when applying to be part of the study.

The three sites who took part were those providing requisite information and agreement to the terms of the study within the recruitment window. ‘Sites’ refers to the geographical areas receiving maternity and early years services provided by the organisations who signed up to the study: Site One involved a local authority and two NHS Trusts (one hospital, one community), Site Two involved two local authorities and one NHS Trust, and Site Three involved one local authority and two NHS Trusts (one hospital, one community). The sites differed in the size of their annual birth cohorts and breastfeeding rates (breastfeeding figures were given for the most recent quarter at the time the sites applied). All three sites had stage 3 UNICEF Baby Friendly hospital accreditation. Details of the sites are summarised in Table 1 below.

**Table 1:** Birth cohort and breastfeeding data for participating sites in 2015

	Site One	Site Two	Site Three
Annual births	3000	4500	10200
Breastfeeding initiation^a^	65.3%	48%	69%
Breastfeeding 6–8 weeks^a^	35.1%	23.5%	50%
UNICEF Baby Friendly accreditation stage	Stage 3 (Hospital Trust) Stage 1 (community)	Stage 3	Stage 3

^a^ Breastfeeding initiation and 6–8 week data given for the most recent quarter in 2015 prior to application to be part of the project

### Parenting resources

Three parenting resources produced by Best Beginnings were evaluated in this study: the Baby Buddy app, Baby Express magazine, and From Bump to Breastfeeding DVD. All three resources were designed to be attractive, easy to use, and provide evidence-based information. The Baby Buddy app, co-created by health professionals and parents, is a complex public health intervention which features a user-designed, interactive avatar (which provides a gaming element), and is designed to provide evidence-based information throughout pregnancy and six months postnatally. It is free to download and is designed to inform parents from all backgrounds, to enhance and augment standard maternity and early years’ service delivery and promote self-care. Baby Express is an age-and-stage baby magazine which has 12 monthly issues. The magazine is designed to be easy to read, and makes extensive use of images. Each issue contains relevant information for that stage of infant development and there is a focus on caregiver–infant relationships. From Bump to Breastfeeding DVD uses real women’s stories to illustrate practical guidance and support for breastfeeding and all its information is now available in the Baby Buddy app.

### Embedding model

Best Beginnings appointed a full-time Regional Facilitator who worked with stakeholders at each of the three sites over a six-month period to design and implement an approach to promote the resources and incorporate their use into antenatal and postnatal care pathways. The approach was underpinned by an ‘Appreciative Inquiry’ philosophy, a philosophy of organisational development that values the positive – what is working well – and emphasises inclusivity and collaborative decision making [15]. It was planned that stakeholders would be encouraged to identify what works well and imagine new possibilities for using the resources in care pathways and relationships with clients. The Regional Facilitator led a series of meetings and workshops for senior management and/or nominated staff members designated as ‘Resource Leaders’. The Resource Leaders were intended to be members of staff from relevant services who were interested in and had the capacity for the role, and who were intended to train their colleagues, and act as champions for the resources and embedding process. Resource Leaders were supplied with materials to support them in training colleagues in the use of the resources: these included a Resource Leaders Cascading Guide, a USB data stick containing a ready-made PowerPoint presentation for use in training, badges and stickers, a booklet guide to the Baby Buddy app, a content guide to the Baby Express magazine, and promotional posters and leaflets. Information about local services was incorporated into the Baby Buddy app and sites were able to customise the inside cover of the Baby Express magazine so that it contained information on local support services. The embedding plan was carried out in each of the three sites in overlapping timescales, with the first embedding workshop (Content and Co-creation) held at Site One in September 2015, Site Two in November 2015 and Site Three in January 2016. Embedding activities ran for six months at each site. A timeline of the key embedding activities, along with data collection activities, is shown in Table 1.

**Fig. 1:**
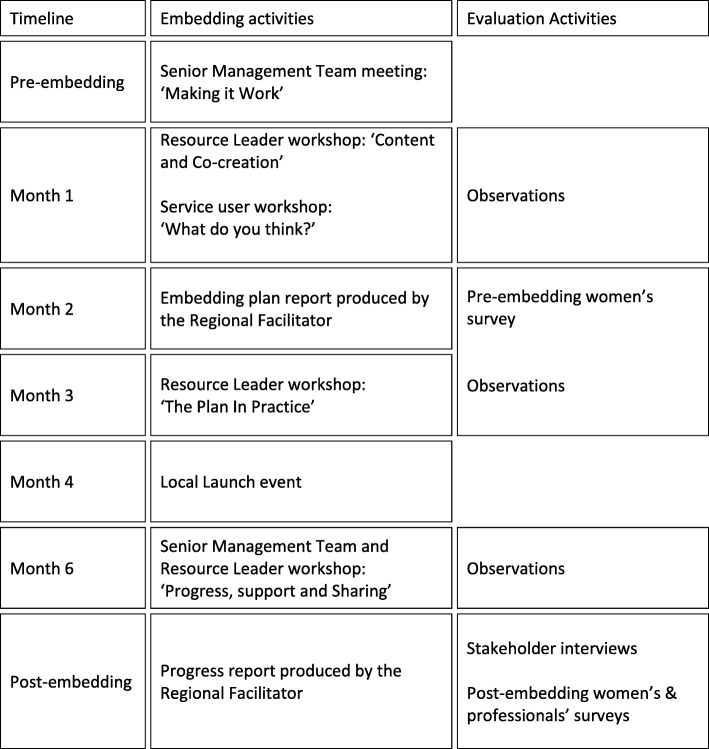
Timeline of the embedding model and evaluation

### Qualitative methods

Observations and interviews were carried out with stakeholders. The stakeholders could be anyone involved with the embedding of the resources: commissioners, health professionals, early years’ professionals, and breastfeeding peer supporters. The ‘Resource Leaders’ described above were the principal stakeholders, along with senior managers in relevant services, and commissioners of services.

#### Observations

We gathered qualitative data through observations at the main embedding activities: the Content and Co-creation workshops, the Plan in Practice workshops (in Site One and Site Three only), and the Progress, Support and Sharing workshops. Participant information sheets were emailed to workshop attendees in advance, to allow time for them to consider participation, and consent was taken at the start of the workshop. Observations aimed to gather information on how participants responded to the resources and the proposed embedding project. They were guided by Spradley’s [16] framework, which describes nine dimensions of social situations. These are space (the physical place where the situation happens), actors (who is involved), acts (specific actions), activity (set of related acts), event (a set of related activities, objects (physical items), goal (what is trying to be achieved), time, and feelings (the emotions being expressed) [16]. Observations were made by the first author, who also participated in the workshops. Observations were made of the room layout and how participants arranged themselves, who participants were (their professions and roles), the aims and activities, interactions occurring between participants, and the views and emotions expressed.

#### Interviews

We conducted telephone interviews with stakeholders in each of the three sites after the final embedding workshop had taken place. We planned to interview approximately 10 stakeholders at each site and to recruit from across the different professional groups involved in the project (commissioners, midwifery, health visiting and Children’s Centres). Potential participants were identified from the consent forms completed when the key embedding workshops were observed, contacted by telephone and/or email, and invited to interview. Snowball sampling was used to identify additional stakeholders who had been involved in embedding activities.

Interviews were semi-structured and aimed to understand participants’ views of, and experiences with, the resources and process of embedding. Participants were asked about their involvement with the project, views on the embedding activities, barriers and facilitators, views of the Best Beginnings resources and perceived impact of the resources, and recommendations for sustainability. The interviews were audio-recorded, lasted between 17 and 50 min and transcribed in full.

#### Documentary review

We also gathered and reviewed the written materials produced by Best Beginnings to support the embedding work, to inform our understanding of the embedding model and its intended implementation. This included regular email newsletters circulated to Resource Leaders in each area by the Best Beginnings Project Coordinator (starting after the Plan in Practice workshop), Embedding and Progress Reports for each area produced by the Best Beginnings Regional Facilitator, and minutes of Senior Management Team (SMT) and/or planning group meetings that occurred as part of the embedding process. Also included were notes from weekly telephone conversations held between the first author and the Best Beginnings Regional Facilitator to discuss the progress of the project at each site.

#### Survey of maternity and early years’ professionals

We designed survey questions (Likert and free text responses) to explore how, and to what extent, the resources had been embedded into maternity care pathways, the level of promotion and use by professionals; and perceived impact on women. Questions aimed to assess the respondent’s awareness of the resources, the uptake and use of the resources, including the extent to which the resources were used within professional–client discussions, views of the resources and recommendations for improvement, and perceptions of the extent to which the resources had been embedded into maternity and early years’ care pathways. We also collected data on the respondent’s age, ethnicity, gender, professional role, length of service and location.

At each site, the survey was issued after the final embedding workshop and professionals in maternity, health visiting and early years’ services, who had contact with pregnant and postnatal women, were eligible to take part. This included both staff members who had attended embedding workshops and those who had not. An online version of the survey was created using the Bristol Online Survey platform, and heads of service or other senior staff disseminated the online survey link to all relevant professionals with a request to participate. Senior managers also distributed paper copies of the surveys if preferred by participants (for example, in some teams the staff completed questionnaires at the end of a team meeting). All paper copies of the survey included a reply-paid envelope for direct return to the evaluation team. Consent to participate was inferred from the return of a completed paper or online questionnaire.

#### Surveys of women

We carried out surveys of women in each of the three sites over a two-month period prior to the launch of the resources, and again for a two-month period after the last embedding workshop. In both phases, women were eligible to participate if they had an infant aged between six weeks to six months (approximately), were aged over 17 years, were able to read/speak English and had no known mental health capacity issues. The pre-embedding survey included questions to explore whether women were aware of or had used the Best Beginnings resources, infant feeding status, and validated scales to assess women’s infant feeding attitudes [17], breastfeeding self-efficacy [18], parenting confidence [19], and mother–infant relationships [20]. Data from the validated scales is presented in a separate paper. In the post-embedding survey these questions were asked again, with additional questions to assess women’s views about the resources and experiences of using them. In both phases, the questionnaires asked for demographic information including age, ethnicity, parity, marital status, educational attainment and employment status.

An information pack comprising survey questionnaire, participant information sheet, and reply paid envelope were distributed to eligible women by health and early years’ professionals. The information packs were distributed at various community locations including health visitor clinics, health visitor visits, baby weighing clinics, and Children’s Centres groups or events. We asked professionals to check eligibility criteria prior to issue, and to provide a verbal explanation of the study and to encourage women to return the survey within the reply-paid envelope once completed. A minimum of 500 survey packs were distributed in each area, at each time point (pre- and post-embedding). In the post-embedding phase, participants were offered a £5 shopping voucher as a ‘thank you’ for their time in taking part. An online version of the survey was created using the Bristol Online Survey platform, and the survey link and information about the evaluation was disseminated via social media connected with local health or Children’s Centre groups. Consent to participate in the survey was inferred from the return of a completed paper or online questionnaire.

### Data analysis

Interview transcripts, observation notes and qualitative data from free-text survey responses were uploaded into MAXQDA 10 qualitative software programme and thematic analysis was conducted based on Braun & Clarke’s [21] approach. Transcripts were read repeatedly, and initial codes developed both inductively from the data and informed by the research questions. Themes were developed iteratively as coding of transcripts progressed, and were derived inductively and with reference to the research questions.

All quantitative data from the surveys were entered into SPSS v22. Descriptive analyses were undertaken for demographic categories (for women: age, ethnicity, marital status, occupation, years of full time education, parity, number of children, age of child; for professionals: age, gender, ethnicity, professional group), and for responses indicating awareness, engagement with, use of, and views of the resources. Where numerical data is presented from the surveys, we have calculated percentages in terms of responses to each question, with missing responses omitted from the total.

Data for the numbers of Baby Buddy app registrations per month for each were supplied by Best Beginnings and reviewed to identify any changes in the numbers of downloads over the course of the embedding work.

### Ethics

Ethical approval for the study was granted by the East Midlands-Nottingham 2 NRES Committee and by the Science, Technology, Engineering, Medicine and Health (STEMH) ethics sub-committee at the authors’ institution (project no. 358). Governance approval was granted by all relevant NHS trusts at each of the three sites. All participants received an information sheet that included the aim and purpose of the study, the voluntary nature of participation, anonymity of the surveys and confidentiality of observations and interviews. The information sheet for the women’s surveys directed women to seek help from a healthcare professional if any of the questions within the questionnaire brought up difficult feelings, and also provided the details for their local Patient Advice and Liaison Service where they could seek further advice and support if necessary. Stakeholders who took part in an observation or telephone interview were asked to provide consent in writing or verbally (i.e. telephone interviews). The Bristol Online survey tool, a secure online platform was used for the online surveys, with all data transferred to password protected/encrypted computer files.

## Results

### Participants

Thirty interviews were undertaken with participants from different professional groups; all were Resource Leaders apart from the commissioners (see Table 2). Where quotations from interviews are reported, participants have been identified by a number and the region is given; however, to preserve anonymity the participant’s professional group is not reported.

**Table 2:** Numbers and types of professionals interviewed

	Site One	Site Two	Site Three
Midwifery	2	2	2
Health visiting	2	2	4
Family Nurse Partnership	1	1	0
Breastfeeding peer support	0	0	1
Children’s centre staff	5	3	4
Commissioning	0^a^	1	1
Total	10	9	11

^a^A new commissioner came into post in Site One during the latter stages of the project and so a decision was made not to request an interview since this individual had had limited experience of the project

Across the three sites, 146 professionals completed a survey. The demographic characteristics of respondents are given in Table 3. Most were aged over 30 years, female, and of White British ethnic origin. Respondents were mostly midwifery, health visiting or children’s centres professionals, with a small number of respondents from neonatal, breastfeeding peer support, or other professional groups.

**Table 3:** Demographic characteristics of professionals’ survey respondents (*n* = 146)

Characteristics	
Age (years)	N (%)
Under 25	1 (0.7%)
25–29	11 (7.5%)
30–34	23 (15.8%)
35–39	16 (11.0%)
40–44	20 (13.7%)
45–49	21 (14.4%)
50–54	25 (17.1%)
55–59	9 (6.2%)
60–64	4 (2.7%)
Missing data	16 (11.0%)
Gender	N (%)
Female	137 (93.8%)
Male	1 (0.7%)
Missing data	8 (5.5%)
Ethnic background	N (%)
White British	135 (92.5%)
White Irish	3 (2.1%)
Black Caribbean	1 (0.7%)
Other Black/Black British	1 (0.7%)
Other ethnic group	1 (0.7%)
Missing data	5 (3.4%)
Professional group	N (%)
Midwifery	30 (20.5%)
Health visiting	49 (33.6%)
Children’s centres	49 (33.6%)
Neonatal	5 (3.4%)
Peer support	2 (1.4%)
Other professional group	7 (4.8%)
Missing data	4 (2.7%)

A total of 353 women completed a survey: 161 in the pre-embedding phase and 192 in the post-embedding phase. The demographic details of respondents to both surveys are shown in Table 4. Most of women who completed the survey at both time points were first-time mothers, aged 30+ years, of white ethnic origin, were either married/civil partnership or living with their partners, stayed in full-time education until 19+ years, and were employed in a paid capacity. There was no significant difference between the pre- and post-embedding samples in the mother’s age, ethnicity, parity, age at which she left full-time education, or infant’s age.

**Table 4:** Demographic characteristics of women’s survey respondents

	Pre-embedding (*n* = 161)	Post-embedding (*n* = 192)
Age (years)		
Under 20	4 (2.5%)	4 (2.1%)
20–24	19 (11.8%)	20 (10.4%)
25–29	39 (24.2%)	49 (25.5%)
30–34	63 (39.1%)	72 (37.5%)
35–39	30 (18.6%)	43 (22.4%)
40 or over	4 (2.5%)	2 (1.0%)
Missing data	2 (1.2%)	2 (1.0%)
Ethnic background		
White British	149 (92.5%)	170 (88.5%)
White Irish	1 (0.6%)	2 (1.0%)
Other white	6 (3.7%)	0 (0%)
Indian	1 (0.6%)	1 (0.5%)
Pakistani	0 (0%)	2 (1.0%)
Black African	0 (0 (0%)	1 (0.5%)
Arab	0 (0%)	1 (0.5%)
Other	0 (0%)	12 (6.3%)
Missing data	4 (2.5%)	3 (1.6%)
Marital status		
Married or in a civil partnership	83 (51.6%)	106 (55.2%)
Living together	54 (33.5%)	56 (29.2%)
Single	16 (9.9%)	26 (13.5%)
Widowed, divorced or separated	1 (0.6%)	0 (0%)
Missing data	7 (4.3%)	4 (2.1%)
Age (years) completed full-time education		
16 or under	14 (8.7%)	33 (17.2%)
17	9 (5.6%)	10 (5.2%)
18	24 (14.9%)	22 (11.5%)
19 or over	105 (65.2%)	119 (62.0%)
Missing data	9 (5.6%)	8 (4.2%)
Occupation		
Studying/training at school, college or university	6 (3.7%)	7 (3.6%)
Working in an unpaid job	0 (0%)	2 (1.0%)
Looking after my family	28 (17.4%)	37 (19.3%)
Not in education, employment or training (because of illness or disability)	0 (0%)	1 (0.5%)
Not in education, employment or training (for other reasons)	3 (1.9%)	5 (2.6%)
Working in a paid job	121 (75.2%)	137 (71.4%)
Missing data	3 (1.9%)	3 (1.6%)
Parity		
First baby	105 (65.2%)	111 (57.8%)
Second or subsequent baby	55 (34.2%)	79 (41.1%)
Missing data	1 (0.6%)	2 (1.0%)
Multiple birth (most recent baby)		
Singleton	157 (97.5%)	185 (96.4%)
Twin	2 (1.2%)	3 (1.6%)
Triplet or other multiple	0 (0%)	1 (0.5%)
Missing data	2 (1.2%)	3 (1.6%)
Total number of children		
1	102 (63.4%)	111 (57.8%)
2	36 (22.4%)	52 (27.1%)
3	10 (6.2%)	19 (9.9%)
4	5 (3.1%)	6 (3.1%)
5	2 (1.2%)	1 (0.5%)
6	0 (0%)	1 (0.5%)
Missing data	6 (3.7%)	2 (1.0%)
Age of child (weeks)		
0–6	12 (8.5%)	22 (11.5%)
7–12	33 (20.5%)	45 (23.4%)
13–18	46 (28.6%)	45 (23.4%)
19–24	44 (27.3%)	39 (20.3%)
25+	21 (13.0%)	36 (18.8%)
Missing data	5 (3.1%)	5 (2.6%)

In the following section we present a synthesis of qualitative and quantitative findings relating to how the embedding model was implemented, how the resources were promoted and distributed, how well they were perceived to fit with care pathways, and women’s and professionals’ views of the resources. Quantitative findings are also summarised in the Additional file 1: Tables S1-S5. An overview of the themes and sub-themes derived from the qualitative data analysis is given in Table 5.

**Table 5:** Overview of the themes of sub-themes derived from the qualitative analysis

Themes	Sub-themes
Implementation of the embedding model	Initiation and “buy in”
	Communication across services
	The Resource Leader role
	Staff training
Promotion and distribution of, and engagement with, the resources	Baby Buddy app awareness
	Baby Express magazine distribution
	From Bump to Breastfeeding DVD awareness
Fit with care pathways	Integration into practice
	Complementing usual care
Perceptions of the resources	Limited appeal for some women
	Reliable and evidence-based
	Visual appeal and readability
	Neutrality and non-judgement

### Implementation of the embedding model

Here we present findings relating to how the embedding model was put into practice. This theme encompasses the sub-themes of ‘initiation and buy in’, ‘communication across services’, ‘The Resource Leader role’, and ‘staff training’. Overall, similar issues were seen at all three sites; however, where an issue was specific to a particular site, this has been made explicit within the text.

Participants reported several factors they believed to be key to successful initiation of the project. Senior management commitment to the project was considered crucial, as it facilitated staff involvement. Participants thought it important to have ‘*sign up from the top’* to ensure this commitment carried through to involve all key staff:

*So yeah, I guess they’re the two things for me that are really facilitated, that we have the staff bought, the service managers bought into this, and then their staff* (Stakeholder interview participant 13, Site Two)

In addition to commitment from senior management, examples were given about how getting colleagues ‘*on board*’ and convinced of the value of embedding in the early stages of the project, helped the process. Here, a stakeholder describes how a staff member who had early reservations about the project was persuaded of the value of the resources and helped to influence colleagues:

*I remember an initial meeting talking about the app and some of the [staff members] were saying to me “well why don’t we just give up our job altogether”, it felt like a bit of a threat really .... What we did with that was to take one of the [staff members] who had those initial concerns and take her on board with the leading of the process, getting her really on board, getting her to look into the app and the magazine and see what was in there and how it could enhance the service that we offer and we found that really helpful in then getting other health practitioners on board.* (Stakeholder interview participant 1, Site Three).

Effective communication between different services was also highlighted as an important facilitator to embedding to promote an integrated strategy to promote and/or deliver the resources, and for stakeholders to learn from each other. The embedding workshops appeared to serve as one avenue for this: each of the workshops was structured to include small group discussions and the first author observed participants sharing experiences and suggestions of what was, or was not, working well. This is also illustrated in the following quote from a stakeholder participant:

*There is a lot of good relationships between health visiting and midwifery anyway, but having the joined-up embedding events, very, very useful rather than it just being maternity, health visiting and children’s centres it was lovely to have that together because you can learn from each other around the pitfalls, well that didn’t work for us this works better, and take things from that.* (Stakeholder interview participant 14, Site Two)

A pivotal component of the embedding model was encapsulated by the subtheme ‘the Resource Leader role’. Resource Leaders were intended to act as champions for the resources and embedding process. At each site, an outline of the Resource Leaders role was circulated to managers within relevant services who were asked to recruit members of their teams. The identification of potential Resource Leaders was managed in various ways, with staff typically being approached if their area of work or training was considered relevant to the project. Some participants reported that the Resource Leader role had been set as an appraisal objective, and this was seen to indicate management support for the work. Participants repeatedly indicated that having motivated, interested Resource Leaders was an important facilitator to ensure smooth embedding. For example, one stakeholder reported:

*I do think it’s the teams who haven’t got, you know, either their Resource Leaders moved on and we’re not aware of it or we haven’t got that person in there that’s sort of leading it, ‘cause in the spaces where we’ve strong Resource Leaders, it just doesn’t seem to be a problem. And I think it’s like that in a lot of the work that we do, we’re relying on somebody to have the passion and the drive to lead the rest of the team*. (Stakeholder interview participant 1, Site Three)

In addition to these views on the personal qualities of Resource Leaders, a frequently mentioned issue was the professional position of the Resource Leaders and how this affected their capacity in terms of time and ability to influence staff practices. Whilst some participants queried giving the role to more senior members of staff who were thought to lack time, others noted that senior staff and those in strategic roles were able to reach and influence more people – ‘*maybe is it seniority over quantity*’. Observations of the embedding workshops uncovered that, at one of the sites, the Resource Leaders from one service were staff at relatively junior grades, who described feeling powerless to influence their more senior colleagues. The overall number of Resource Leaders in a service also had an impact on their effectiveness with some services having a relatively small number of Resource Leaders to cover a large area:

*The only drawback that me and [colleague] have found is that we cover a large area so to get round every centre and every member of staff, that has been difficult, we would have hoped really that more members of staff were trained [as Resource Leaders].* (Group Stakeholder interview participants 4 & 30, Site Two).

The final subtheme in this theme was ‘staff training’. After the selection and training of Resource Leaders, the next stage of the embedding process was for Resource Leaders to cascade training to wider staff within their service. As described in the outline of the embedding model, staff training was supported with material supplied by Best Beginnings. Several participants, as described in the quote below, valued having the ready-made PowerPoint presentation as it provided a structure for them to use in training sessions:

*The USB sticks with the PowerPoint on – that was a really good idea because that meant obviously I didn’t have to go back and then try and figure how I was going to train up the staff because it was all there for you, it was brilliant. We just got together in team meetings literally plugged in the stick and then this allowed us to go through each slide and then print off anything for practitioners that they can then keep to take out*. (Stakeholder interview participant 21, Site One)

Other participants described using sections of the presentation rather than delivering it in its entirety. They chose segments based on what they felt was most relevant to their particular context and colleagues, and what time allowed. In some cases, pressures on staff time meant that ‘*we put on a lot of sessions but no one came*’ and Resource Leaders developed alternative approaches to reach staff, such as brief informal information-giving time around shift changes:

*At the beginning of shift when they had their handover you know, patients allocated etc. You’d go in sort of 10 minutes afterwards*. (Stakeholder interview participant 26, Site One)

As well as providing initial training and familiarisation of the resources, Resource Leaders also took on the role of maintaining staff awareness and keeping the use of the resources at the forefront of people’s minds. Several participants across the sites described achieving this through having the project as a standing agenda item at team meetings to ensure it was regularly revisited:

*Everybody has said really, and we have as a staff team, we have a staff meeting once a week on either a Tuesday or a Thursday, and we’ve actually got them out and so that we don’t get stale, we still get them out and we look through them and then we tend to look so we do the outreach visits and do those, we just, if we keep up to date, and whenever we’ve got five minutes we’ll have a read through just so that we’re making sure that our families are getting the best from them*. (Stakeholder interview participant 10, Site Three)

Best Beginnings provided ongoing support throughout the project in a number of ways. The Regional Facilitator was available to be contacted by Resource Leaders and SMT at each site to help troubleshoot any issues arising. Resource Leaders were sent ‘Resource Leader Bulletins’: these were monthly support emails from Best Beginnings giving progress updates of the project, reminders to complete the staff training log sheets, and reminders to supply any new local information for integration into the Baby Buddy app).

### Promotion and distribution of, and engagement with, the resources

This theme encompassed the sub-themes ‘Baby Buddy app awareness’, ‘Baby Express magazine distribution’ and ‘From Bump to Breastfeeding DVD awareness’.

#### Baby buddy app

Promotion and publicity was felt to be an important component of the embedding work, particularly with regard to the Baby Buddy app. Participants made use of the promotional materials that were supplied by Best Beginnings, for example making extensive use of posters to raise awareness of the app:

*There’s a poster up in every room. Everywhere you look there’s a Baby Buddy app poster up, in the toilets, they’re everywhere, yeah, they’re everywhere. We couldn’t display it any more than what we’ve got it displayed as.* (Stakeholder interview participant 28, Site One)

Leaflets and flyers advertising the app were also widely used, either being given to women at appointments or posted with booking appointment letters. Participants also described talking about the Baby Buddy app in their contacts with women, and views varied as to the perceived feasibility of this. For some participants, introducing the app was seen to be achievable within a normal contact:

*The thing is it is brief, it only takes a few minutes to mention the app and talk about the app and the message is across there. A lot of people like their phones and use their phones, mums do.* (Stakeholder interview participant 15, Site Two)

Other professionals referred to the time constraints they were under at appointments, and did not consider it realistic to introduce the app, particularly at the booking appointment:

*Once we see them in their booking there is* so *much info to give them/paperwork to fill out it is not the priority to promote this.* (Professionals survey 21, Site One)

The Baby Buddy app was available free to download throughout the UK prior to the embedding work. Overall, the survey responses and registration data collected from the app indicated that substantially more women had downloaded the Baby Buddy app in the months after the embedding process had started compared with prior to embedding activities. Data supplied by Best Beginnings showed that there were 10 Baby Buddy app registrations in Site One in August 2015, the month prior to the first embedding activity. In Sites Two and Three in the equivalent months (October 2015 and December 2015) there were 21 and 18 registrations respectively. In the month after the local launch event at each site, the numbers of registrations were 212 at Site One (January 2016), 233 at Site Two (March 2016), and 685 at Site Three (June 2016), the greater number at Site Three reflecting the larger birth cohort. In the post-embedding women’s survey, 75% (143/192) had heard of the Baby Buddy app. Of those who had heard of the app, overall across the three sites, about three-quarters had had a conversation with a professional when first finding out about the app (Additional file 1: Table S1).

Some professionals noted that the Baby Buddy app registration data at ward level indicated that the app was being downloaded across the area including parts of the region where it was perceived to be ‘harder to reach’ women:

*So obviously we would adapt our approach anyway but that app reinforces another way and it was interesting looking at the data that some of our harder-to-reach areas such as* [named area] *and* [named area] *are really using that app well and they are some of our much harder-to-reach places. So it means they are not only using us but are using another resource for information which supports their caring of their child.* (Stakeholder interview participant 24, Site One)

#### Baby express magazine

The Baby Express magazine has 12 issues, each issue designed to be appropriate for a baby of a particular month of age, with issue one aimed at parents of babies aged up to one month, and so on. Participants frequently said that postal delivery of the Baby Express magazine on a monthly basis, so that women received the magazine at the right time, would have been ‘*ideal*’. However, the cost implications were too great for this to be a feasible option. There was also the consideration of how delivery of the magazines could be sensitively managed so that in the case of infant loss, women did not continue to be sent issues of the magazine. Given these limitations, all three sites opted to tie Baby Express delivery schedules to professionals’ contacts with women. However, the low number of universal contacts with professionals meant that magazines had to be given well in advance of the infant’s age. For example, issues three and four (for babies aged three months and four months respectively) were given at the 10-day postnatal appointment in Site One, and at the six-to-eight-week appointment in Sites Two and Three.

In the post-embedding women’s survey, across the three sites, 53% of women had received a copy of the Baby Express magazine, suggesting that issues of the magazine were not as widely received as hoped (Additional file 1: Table S1). This is likely due to the logistical problems with the delivery encountered at all three sites. Participants frequently referred to the difficulties of delivering the magazines to women, and of coordinating the delivery across different services, as expressed here:

*Something needs to happen, like coming together and arrangement made between the health visitor teams and the midwives and the children’s centre to ensure that flow is actually happening, because otherwise like I said before, the magazine and the whole idea of it is not working. Obviously the idea is that if they get every issue, they get everything that they can out of every issue, but at the moment it is not happening.* (Stakeholder interview participant 21, Site One).

All three sites elected to distribute the later issues of the magazines via Children’s Centres (Site One did this from Issue 5 onwards and Sites Two and Three from Issue 7 onwards), since at this stage women did not have routine contacts with midwifery or health visiting staff. This strategy relied on professionals at earlier contacts advising women to go to their local Children’s Centre to collect the later issues of the magazines, and on women themselves opting to go to the centre to collect the magazine. Some stakeholders expressed concerns about whether this would be an effective dissemination strategy:

*We were presuming that the health visitors after they’d given issue four would say “pop into your children centre to pick your next copy up” but then it depends how active and engaged that parent is to think I need my next copy of my magazine let me pop to my children’s centre. In reality that may not happen*. (Stakeholder interview participant 7, Site Two).

In targeted services, for example those serving more vulnerable women, the higher number of regular staff contacts streamlined the process of giving issues of the magazine:

*In our programme we don’t have an issue of handing them out because we see them frequently anyway so we not having to go out and do extra visits to get them delivered, we’re already going anyway.* (Stakeholder interview participant 2, Site Two)

Most women who received a copy of the magazine had engaged with the magazine to some extent, with only 13% (13/101) of respondents to the women’s survey reporting that they had not read any information in any magazines they had received.

#### From Bump to Breastfeeding DVD

The ‘From Bump to Breastfeeding’ DVD was intended as a back-up resource for parents who either could not access the Baby Buddy app, or whose first language was one of the languages available on the DVD (Arabic, Urdu, Bengali, Polish, Somali, and British Sign Language). Of the 191 women who responded to the question in the post-embedding survey, 61 had received a copy of the DVD (Additional file 1: Table S1). Professionals reported using the DVD with women only rarely, because they worked with very few women who did not have English as a first language, and/or because most women they worked with were able to access the Baby Buddy app:

*The mums we do use it with are few and far between, with English not as their first language. So I have used it a few times but certainly not that often*. (Stakeholder interview participant 26, Site One).

Of the 61 women who had received a copy of the From Bump to Breastfeeding DVD, 31 had watched it in English, one woman had watched it in Bengali, and the remainder did not answer this question.

### Fit of the resources with care pathways

Two sub-themes – ‘integration into practice’ and ‘complementing usual care’ – comprised this theme. The embedding process was based on the idea that the resources would be integrated within existing care pathways. This was seen as key to the project being accepted by staff, as it helped the resources to be seen as manageable. One participant reported, in relation to the Baby Buddy app:

*I think it’s been received well by staff because it’s so easy to use the resources, because it fits in well with our care pathway, our booking appointments. We do talk about where to get information from so it’s a good opening into giving the leaflet that explains about the app, so I think it’s been received by the staff and there hasn’t been any negativity about it being an added pressure*. (Stakeholder interview participant 6, Site Three)

Conversely, other participants, as reflected in the quote below reported that staff found it hard to sustain use of the app in their day-to-day work, given the multiple competing tasks they had to fulfil:

*I just do think it could be used more than it is; however, our staff would say there’s that many other resources that they’ve got to use within the work that it’s really difficult to keep that at the forefront.* (Stakeholder participant interview 7, Site Two)

In the professionals’ survey, 83 of 141 respondents (59%) felt that the Baby Buddy app was ‘very much’ or ‘quite a lot’ embedded into practice (Additional file 1: Table S2). Across the three sites, 59 of 125 professional survey respondents (47%) said that they referred to the app with women ‘frequently’. A few participants described feeling that the Baby Buddy app ‘*is like part of us now’* and talked about how they felt that using the app had become a standard part of their practice:

*We don’t have to think extra about it, it’s just part and parcel … it’s like what you would do when you engage with people*. (Group Stakeholder interview participants 4 & 30, Site Two).

Underpinning the embedding plan was the idea that the resources were to complement rather than substitute health professionals’ contacts with women. Some professionals in the survey and interviews were at pains to emphasise this point:

*I don’t feel either resource will help with relationships with baby and partner or discussions with health professionals. Relationships depend on the individuals and health professionals have always had meaningful discussions – if they don’t, they shouldn’t be in the profession.* (Professionals survey 122, Site Three)

Relatedly, a few respondents to the women’s post-embedding survey reported that they preferred face-to-face help rather than seek information from an app:

*No time for this type of thing. Would rather go to a baby/breastfeeding group and talk in person*. (Women’s post-embedding survey 94, Site Two)

Fifty-four of 141 (38%) respondents in the professionals’ survey felt that Baby Express was ‘very much’ or ‘quite a lot’ embedded into practice, probably reflecting the logistical difficulties with the distribution of the magazine. With regard to the From Bump to Breastfeeding DVD, 39 of 138 (28%) respondents felt the DVD was ‘very much’ or ‘quite a lot’ embedded into practice, perhaps reflecting the supplementary role this particular resource was intended to play (Additional file 1: Table S2).

In the professionals’ survey, respondents were asked to indicate the contexts in which they would use the Baby Buddy app with women. Just under half of respondents overall (72/146; 49%) indicated that they used the app at antenatal appointments, 65 of 146 (45%) at mother and baby groups, and 59 of 146 (40%) at parent education classes (Additional file 1: Table S3; note that respondents could choose more than one response). One participant described how women were encouraged to use the Baby Buddy app by offering suggestions at parent education classes on aspects of the app to explore:

*Colleagues who deliver our parenting courses… are finding that although some of the women have got the app, that they’re helping them to explore it in different ways and saying can you have a look at this on the app for next week and tell me something about it when you arrive, but not always easy for all women, but it’s helping women to delve a little bit deeper into things and look for specific things.* (Stakeholder interview participant 17, Site Three)

In the professionals’ survey, mother and baby groups were reported to be a popular context for professionals to use the Baby Express magazine (61/146; 41.8%). Postnatal appointments and parent education classes were also highly cited (53/146; 36.3% each. Respondents could choose more than one response to this question. See Additional file 1: Table S3.) In some Children’s Centres in Site Two, all staff had been trained to use the Baby Express magazines, and copies of the magazines were available in the reception area, so that if parents asked questions, they could refer to the magazines:

*We’ve actually trained all the staff, so staff know that they are in reception, that they can go to them and that they can refer to them, like [staff name] said if they’ve got a question around colic they know which magazine to go to, which page to go to, and then the information is there for them*. (Group Stakeholder interview participants 4 & 30, Site Two)

Some participants reported how they used the magazines with women on home visits, and structured the interaction with the women around the information contained within the magazines. One stakeholder stated:

*I don’t hand them over at the beginning of the visit I actually physically use them as my introduction to the health promotion activities that we discuss. So I actually open up the page and we talk about the communication, the attachment section, how babies learn section, the cot safety section, the feeding section so I use each section to then talk about each of those health promotion activities with the client so that I am actually using the book as my means of while I am talking about it with the clients, and then they can look at it and they can ask questions if they have got any at the same time, so we do it together*. (Stakeholder interview participant 27, Site Three)

To aid embedding work, Best Beginnings provided sites with a Baby Express content guide, which was an index to locate topics in the issues of the magazine. Some participants described using this to ‘*map*’ magazine content to the particular programmes their service offered; this then gave them a ready reference to access relevant information during the session and to share with parents:

*So we mapped everything that we do against what was in the magazine. So number nine on the back we put baby sign in the sensory room and that was all about learning to talk and stuff like that, and then number 10 we put – have you heard of heuristic play? – ‘cause in issue 10 it talks about making toys out of household objects. So that fit beautifully with heuristic play.* (Stakeholder interview participant 29, Site One)

In the professionals’ survey, 69 (47%) of 146 respondents reported using the From Bump to Breastfeeding DVD at antenatal appointments, and 58 (39.7%) in parent education classes (Additional file 1: Table S3). Several respondents related that the DVD was often played in the waiting area of their service:

*This DVD is very good and played regularly in the children’s centres whilst parents wait for antenatal appointments*. (Professional survey 26, Site One)

### Women’s and professionals’ perceptions of the resources

This theme comprised the sub-themes of ‘limited appeal for some women’, ‘reliable and evidence-based’, ‘visual appeal and readability’ and ‘neutrality and non-judgement’. Overall, 69% (in the post-embedding women’s survey; 81/117; Additional file 1: Table S4) of women and 92% of professionals (128/138; Additional file 1: Table S5) rated the quality of the Baby Buddy app as ‘excellent’, ‘very good’, or ‘good’. Women typically identified the information within the app as particularly useful, and made reference to the reassurance or increased confidence they gained. However, some of the women survey respondents felt the app did not provide enough detailed information, or felt that the information was tailored to first-time mothers, and a few described the avatar, and the gaming elements of the app, as ‘*childish’*. A few professionals, as reflected below, also perceived the avatar to hold limited appeal to older women:

*I think it’s more suitable probably for the younger mums, that’s sort of the, from the feedback that we’ve got. They like the videos and things but in terms of making an avatar of yourself, the mums that are in their thirties aren’t really particularly keen on doing that, you know.* (Stakeholder interview participant 19, Site One)

Some professionals appreciated that the information contained in the app could be relied upon to be evidence-based so that they could feel confident in recommending the app to women; this was particularly valued when it was considered that many women had downloaded one or more other apps whose reliability was uncertain:

*A few of them have got different apps, I think some, they find out they’re pregnant and they download everything and want to know everything and I just sell it as this is the only one that is evidence-based, it’s not people’s opinions, because people’s opinions, in the best will in the world think they could be helping you when really it’s their experience, it’s their app, you know, opinion*. (Stakeholder interview participant 9, Site Three)

Presenting information in video format was frequently cited as a positive feature of the app, with staff describing how having access to video information helped them support women in the home environment:

*I found that the footage within the app around infant feeding, specifically the breastfeeding, there was some useful footage in there that I could use when I was trying to explain to a mum with a complex feeding problem around positioning and attachment, and to be fair that is the only part of the app that I actually used. I found it so useful in a home environment where you are trying to explain something and you need something visual*. (Stakeholder interview participant 14, Site Two)

Seventy-seven percent of women (72/94; Additional file 1: Table S4) and 86% (116/135; Additional file 1: Table S5) of professionals rated the quality of Baby Express magazine as ‘excellent’, ‘very good’, or ‘good’. Several women commented on how the discrete age-and-stage nature of information presented in the Baby Express magazine meant that the material was easier to access and more manageable to absorb:

*Useful to skim read as it’s not too in-depth. Easy to dip in and out of the sections that are most useful. Like that they're categorised by age so info is very relevant.* (Women’s post-embedding survey 16, Site One)

The magazine’s focus on relationships was mentioned by several respondents as a useful feature of the magazine, who welcomed the ‘*ideas for play*’ and ‘*tips*’ for ways of engaging with their baby:

*How baby is developing at each month and ideas of how to interact with baby at the stage they are at. Brilliant magazines!* (Women’s post-embedding survey 3, Site One)

Similarly, professionals considered the magazines to be particularly useful in supporting relationship-building activities, due to both their content on child development and emphasis on responsive caregiving:

*The magazines are excellent, they are informative and easy to understand. Such as bedtime routines, breastfeeding and solids and play ideas, which are great for bonding and child development*. (Professionals survey 108, Site Two)

Some participants considered that the magazines provided a way of imparting information without parents feeling judged or criticised for previous choices. Participants spoke of the importance of not judging parents and how it could sometimes feel difficult to give information that was contrary to parents’ usual practices. The magazines were seen to be a neutral yet authoritative source of information that resolved this issue:

*One of the parents that read it she said, there’s a heading that says ‘Still too young for food’ or ‘Still too young for solids’, well she’d remembered that and then actually where she’d looked the fact, this is a mum who’d got a four month old and she’s said “oh no, no, no she can’t have it yet, I remember reading it” and she read through the whole thing, she read me this saying so too young for solids or still too young for food and she smiled and she didn’t feel like she was being demonised. She was just thinking that maybe she’s a little bit young to be, I’m going to wait a little bit more, and that then helped her when she looked at you know the baby-led weaning because it were information that she’d get*. (Stakeholder interview participant 10, Site Three)

As a supplementary resource, the From Bump to Breastfeeding DVD was less used than the other resources, and much less discussed in interviews. However, among those who did use it, 71% (24/34; Additional file 1: Table S4) of women agreed or strongly agreed that the DVD was easy to use and easy to understand. Similar views were reported by professionals, with 70% (82/117) and 72% (85/117) of respondents either strongly agreeing or agreeing that the DVD was easy to use and easy to understand respectively (Additional file 1: Table S5).

## Discussion

Our evaluation identified some key barriers and facilitators to the embedding of the resources into care pathways. Important facilitators included early, active engagement from senior managers and staff at team leader level, and having a sufficient number of Resource Leader champions to cover both the geographical area and the number of staff needed to be trained in each service. To be effective, Resource Leaders needed to be motivated and enthusiastic and to “take ownership” of the project, to have enough seniority to be credible among the staff they were training, and to have the backing of senior managers. Several approaches to training were employed to reach busy professionals, with Resource Leaders using specially scheduled training sessions, existing team meetings, and delivering short bursts of information to staff at shift changeovers or while on clinical shifts. Keeping the resources “on the agenda” via email updates and team meetings, and widespread publicity, were considered key components of maintaining awareness and engagement with the resources. Embedding work greatly increased the number of downloads of the nationally available Baby Buddy app at each of the three sites. Distribution of the Baby Express magazine was problematic at all three sites due to the limited number of staff contacts with women. Professionals reported struggling with time pressures when trying to train colleagues or to incorporate use of the resources into their practice. The resources were generally well received by women and health professionals: they were seen as easy to use, appealing, and informative, although some participants expressed a wish for more in-depth information or felt they would be unlikely to make extensive use of them.

Our study was conducted across three sites giving comparative data on how the embedding model was implemented and how – and the extent to which – embedding of the resources was achieved in different settings. The professional survey was completed by a high number of respondents and the in-depth semi-structured interviews with key stakeholders provided rich data on the embedding process, use of the resources in practice, and barriers and facilitators to embedding. The timing of the evaluation meant that we were only able to capture the early stages of the embedding process: while this is valuable in itself, the results must be interpreted with caution. For example, the scope of the project meant that we could not capture the later stages of Baby Express delivery, and therefore cannot report on whether the delivery method of women collecting from the Children’s Centres was effective. Because the survey was disseminated via a variety of email mailing lists and via paper questionnaires, we do not have a precise denominator and so a response rate cannot be calculated. We do not have data on how respondents to the professionals’ survey compared to those who did not complete the survey, and it is possible that professionals who were less engaged with the project did not participate. Similarly, as qualitative participants were recruited through the stakeholder workshops and via snowball sampling, we may be missing views of those who were less engaged. A further limitation is the low response rate to the women’s surveys, and the fact that our sample was composed mainly of women who were over 30 years of age, described their ethnicity as White British, were in paid employment and had stayed in full-time education past the age of 19 years. The scope of the current study did not include qualitative research with women, but this would have given further valuable insights into women’s views, usage and experiences with the resources.

Many of our findings echo those of other studies examining the use of digital health applications in maternity care. For example, in this study, and in the studies by Goetz et al. [8] and Wilcox et al. [7], participants expressed views that mHealth and eHealth resources should complement rather than replace face-to-face information from health professionals: we found similar views from both women and professionals. Stapleton and colleagues [13] reported on the barriers to the effective integration of evidence-based leaflets into maternity care and found that time pressures on professionals were a hindrance to integrating information tools into practice. In our study, staff gave mixed views, with some finding time pressures prohibitive particularly at busy appointments, while others reported being able to incorporate the resources into their contacts. In the study by Stapleton et al., observations of interactions between women and midwives found that midwives did not draw attention to or discuss the leaflets with women. While we do not have observational data of women–professional interactions, our survey and interview data from both women and professionals indicate that professionals were discussing the resources with women. Stapleton et al.’s study also reported low engagement of women with the leaflets; we found variable engagement of women with the resources. Baby Buddy app registrations greatly increased after embedding activities, and in the survey, most women who had received a copy of Baby Express magazine had read at least some of the content, but issues with delivery meant not all eligible women had received a copy of the magazine, and few women received a copy of the From Bump to Breastfeeding DVD.

Our study has some implications for how health services might plan to incorporate supportive health promotion and education tools into care pathways to support new parents. Careful consideration should be given to how points of contact with women can be best used to deliver material resources such as magazines or DVDs, to ensure that these actually reach women and to maximise the chance of women engaging with the resource. Similarly, careful consideration should be given about how to give information about, and demonstrate and encourage the use of, digital resources. Commitment from senior managers and team leaders is needed to ensure embedding is seen as a priority by staff, and this priority needs to be sustained through regularly revisiting embedding work at team meetings and ongoing promotion and publicity. Staff who are using the resources should receive regular feedback about resource usage (for example, data gathered by the digital applications) to help maintain motivation.

## Conclusion

Overall, our findings suggest that incorporating supportive parenting resources into maternity and early years’ care pathways requires a planned embedding approach supported and promoted by committed champions and senior management. There are logistical issues with linking resource delivery into care pathways where staff have limited contacts with women, and time pressures present a barrier. Initial findings indicate largely positive views of women and professionals and suggest the resources can be a beneficial aid for pregnant women and new mothers.

## Additional file

Additional file 1: **Table S1.** Women’s awareness of, receipt of and engagement with the resources (post-embedding). **Table S2.** Professionals’ perceptions of extent to which resources were embedded into practice. **Table S3.** Professionals’ reports of context of use of the resources. **Table S4.** Women’s rating of the quality of the resources (post-embedding). **Table S5.** Professionals’ rating of the quality of the resources. (DOCX 18 kb)

## Data Availability

Data from this study is not available, in order to protect participant confidentiality.

## Abbreviations

NHS: National Health Service
SMT: Senior Management Team
