# Supplementary material for: Embedding supportive parenting resources into maternity and early years care pathways: a mixed methods evaluation
Source: BMC Pregnancy Childbirth. 2019 Jul 22;19:253. doi: 10.1186/s12884-019-2388-2 (PMC6647328; doi:10.1186/s12884-019-2388-2)
Supplement: Supplementary file 1 — Table S1. Women’s awareness of, receipt of and engagement with the resources (post-embedding). Table S2. Professionals’ perceptions of extent to which resources were embedded into practice. Table S3. Professionals’ reports of context of use of the resources. Table S4. Women’s rating of the quality of the resources (post-embedding). Table S5. Professionals’ rating of the quality of the resources. (DOCX 18 kb) [file 12884_2019_2388_MOESM1_ESM.docx]

**Table S1: Women’s awareness of, receipt of and engagement with the resources (post-embedding)**

| Women’s post-embedding survey | | | N=192 |
| --- | --- | --- | --- |
| Baby Buddy app | Aware of Baby Buddy app | Yes | 143 (75%) |
|  |  | No | 49 (25%) |
|  | Discussion of Baby Buddy app with professional | Yes | 109 (57%) |
|  |  | No | 34 (18%) |
|  |  | Not applicable (unaware of the app) | 49 (25%) |
| Baby Express magazine | Received a copy of Baby Express magazine | Yes | 103 (53%) |
|  |  | No | 86 (45%) |
|  |  | Missing data | 3 (2%) |
|  | Discussion of Baby Express with a professional | Yes | 83 (43%) |
|  |  | No | 16 (8%) |
|  |  | Missing data | 7 (4%) |
|  |  | Not applicable (no receipt of magazine) | 86 (45%) |
| From Bump to Breastfeeding DVD | Received a copy of or information about the DVD | Yes | 61 (32%) |
|  |  | No | 130 (67%) |
|  |  | Missing data | 1 (1%) |
|  | Discussion of DVD with a professional | Yes | 38 (20%) |
|  |  | No | 18 (9%) |
|  |  | Missing data | 5 (3%) |
|  |  | Not applicable (no receipt or information about DVD) | 130 (68%) |

**Table S2: Professionals’ perceptions of extent to which resources were embedded into practice**

|  | Baby Buddy app  (n=146) | Baby Express magazine  (n=146) | From Bump to Breastfeeding DVD  (n=146) |
| --- | --- | --- | --- |
| Very much  Quite a lot  Unsure  A little  Not at all  Missing data | 33 (22.6%)  50 (34.2%)  30 (20.5%)  25 (17.1%)  3 (2.1%)  5 (3.4%) | 20 (13.7%)  34 (23.3%)  52 (35.6%)  21 (14.4%)  14 (9.6%)  5 (3.4%) | 7 (4.8%)  32 (21.9%)  59 (40.4%)  24 (16.4%)  16 (11.0%)  8 (5.5%) |

**Table S3: Professionals’ reports of context of use of the resources**

|  | Baby Buddy app  n=146 | Baby Express magazine  n=146 | From Bump to Breastfeeding DVD  n=146 |
| --- | --- | --- | --- |
| Parent education  Booking appointment  Antenatal appointment  Clinic contact  Postnatal appointment  Baby Massage  Mother and baby group  Breastfeeding group  Other | 59 (40.4%)  35 (24.0%)  72 (49.3%)  50 (34.2%)  56 (38.4%)  35 (24.0%)  65 (44.5%)  52 (35.6%)  19 (13.0%) | 53 (36.3%)  15 (10.3%)  49 (33.6%)  40 (27.4%)  53 (36.3%)  30 (20.5%)  61 (41.8%)  40 (27.4%)  21 (14.4%) | 58 (39.7%)  15 (10.3%)  69 (47.3%)  27 (18.5%)  37 (25.3%)  22 (15.1%)  41 (28.1%)  39 (26.7%)  18 (12.3%) |

Respondents could choose more than one answer

**Table S4: Women’s rating of the quality of the resources (post-embedding)**

|  | Baby Buddy app  n=143^a^ | Baby Express magazine n=103^a^ | From Bump to Breastfeeding DVD  n=61^a^ |
| --- | --- | --- | --- |
| Excellent  Very good  Good  Okay  Poor  Very poor  Missing data | 20 (14.0%)  31 (21.7%)  30 (21.0%)  32 (22.4%)  2 (1.4%)  2 (1.4%)  26 (18.2%) | 16 (15.5%)  32 (31.1%)  24 (23.3%)  21 (20.4%)  1 (1.0%)  0 (0%)  9 (8.7%) | 5 (8.2%)  9 (14.8%)  9 (14.8%)  12 (19.7%)  1 (1.6%)  0 (0%)  25 (41.0%) |

a: The total number of respondents represents the number who answered ‘yes’ to having heard of or received each resource

**Table S5: Professionals’ rating of the quality of the resources**

|  | Baby Buddy app  n=146 | Baby Express magazine  n=146 | From Bump to Breastfeeding DVD  n=146 |
| --- | --- | --- | --- |
| Excellent  Very good  Good  Okay  Poor  Very poor  Missing data | 44 (30.0%)  48 (32.9%)  36 (24.7%)  6 (4.1%)  2 (1.4%)  2 (1.4%)  8 (5.5%) | 37 (25.3%)  48 (32.9%)  31 (21.2%)  15 (10.3%)  2 (1.4%)  2 (1.4%)  11 (7.4%) | 13 (8.9%)  42 (28.8%)  48 (32.9%)  16 (11%)  1 (0.7%)  2 (1.4%)  24 (16.4%) |
